# Optimized saturation pulse rrains for SASHA T_1_ mapping at 3T

**DOI:** 10.1186/1532-429X-17-S1-W20

**Published:** 2015-02-03

**Authors:** Kelvin Chow, Peter Kellman, Bruce S Spottiswoode, Sonia Nielles-Vallespin, Richard B Thompson

**Affiliations:** 1 University of Alberta, Edmonton, AB, Canada; 2 NIH, Bethesda, MD, USA; 3 Siemens Healthcare, Chicago, IL, USA

## Background

SASHA and MOLLI T_1_ mapping sequences can have errors in calculated T_1_ values when their magnetization preparation pulses do not fully saturate/invert magnetization [1,2]. The commonly used 90°-90°-90° saturation pulse train [3] has poor performance at 3T due to large B_1_ field inhomogeneities. We propose that a new hard RF pulse train with numerically optimized flip angles [4] will offer superior performance and reduce errors in SASHA T_1_ values due to incomplete saturation.

## Methods

Flip angles for a 6-pulse train were optimized by minimizing the maximum residual longitudinal magnetization in Bloch equation simulations performed over ranges of values expected at 3T: 40-120% B_1_ scaling, -240-240 Hz off-resonance, 200-2000 ms T_1_, and 14 μT B_1_ strength. Complete spoiling of transverse magnetization was assumed during spoilers. Optimization code is available at https://bitbucket.org/kelvinc/pulsetrainopt.

Saturation performance for the 90°-90°-90° and the 6-pulse train was measured in a phantom with saturation recovery GRE. B_0_ and B_1_ maps were calculated using multi-TE and multiple flip angle GRE respectively. A magnetic field gradient was used to produce a range of off-resonance and experiments were repeated with the prescribed pulse train flip angles scaled by 40-120% to emulate B_1_ inhomogeneity.

SASHA and MOLLI T_1_ mapping were performed using investigational prototype sequences on a 66 kg swine in a 3T system (MAGNETOM Skyra, Siemens AG, Germany). SASHA was acquired using both the 90°-90°-90° and proposed pulse train with a 45° imaging flip angle. MOLLI used an optimized inversion pulse (2) with a 20° flip angle. A B_1_ map was acquired using a saturated double angle method with single-shot EPI readouts.

## Results

**Figure 1:**
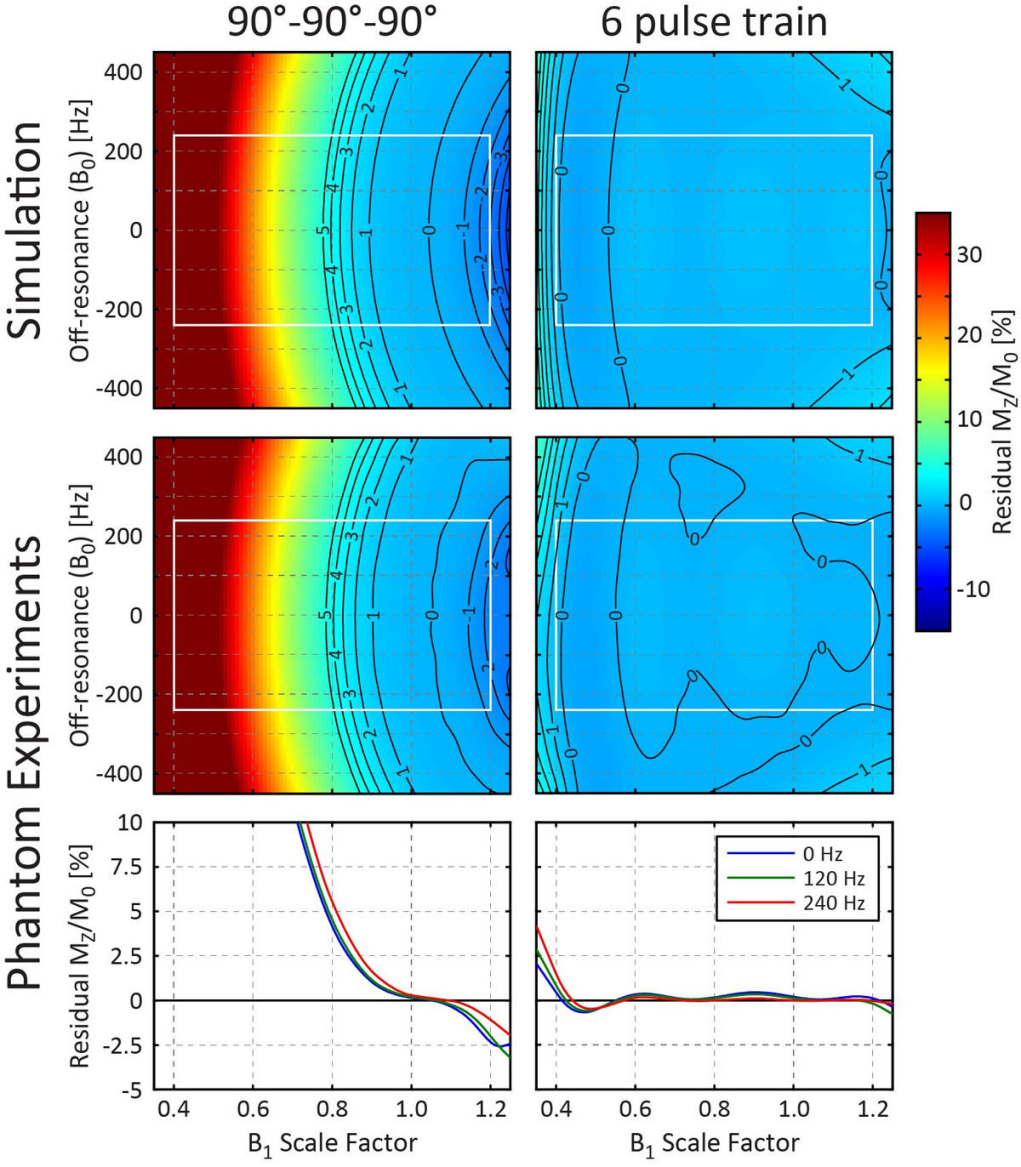
Simulated and experimentally measured residual longitudinal magnetization for a commonly used 90°-90°-90° saturation pulse train and a proposed 6 pulse train. White boxes denote the 3T optimization range.

The optimized 6-pulse train flip angles were 115-90-125-85-176-223° with a 33 ms duration. The 6-pulse train had excellent performance (Fig. 1), with an average and maximum absolute residual longitudinal magnetization over the optimization range of 0.27% and 0.87% respectively. Experimental data had excellent agreement with simulations.

**Figure 2:**
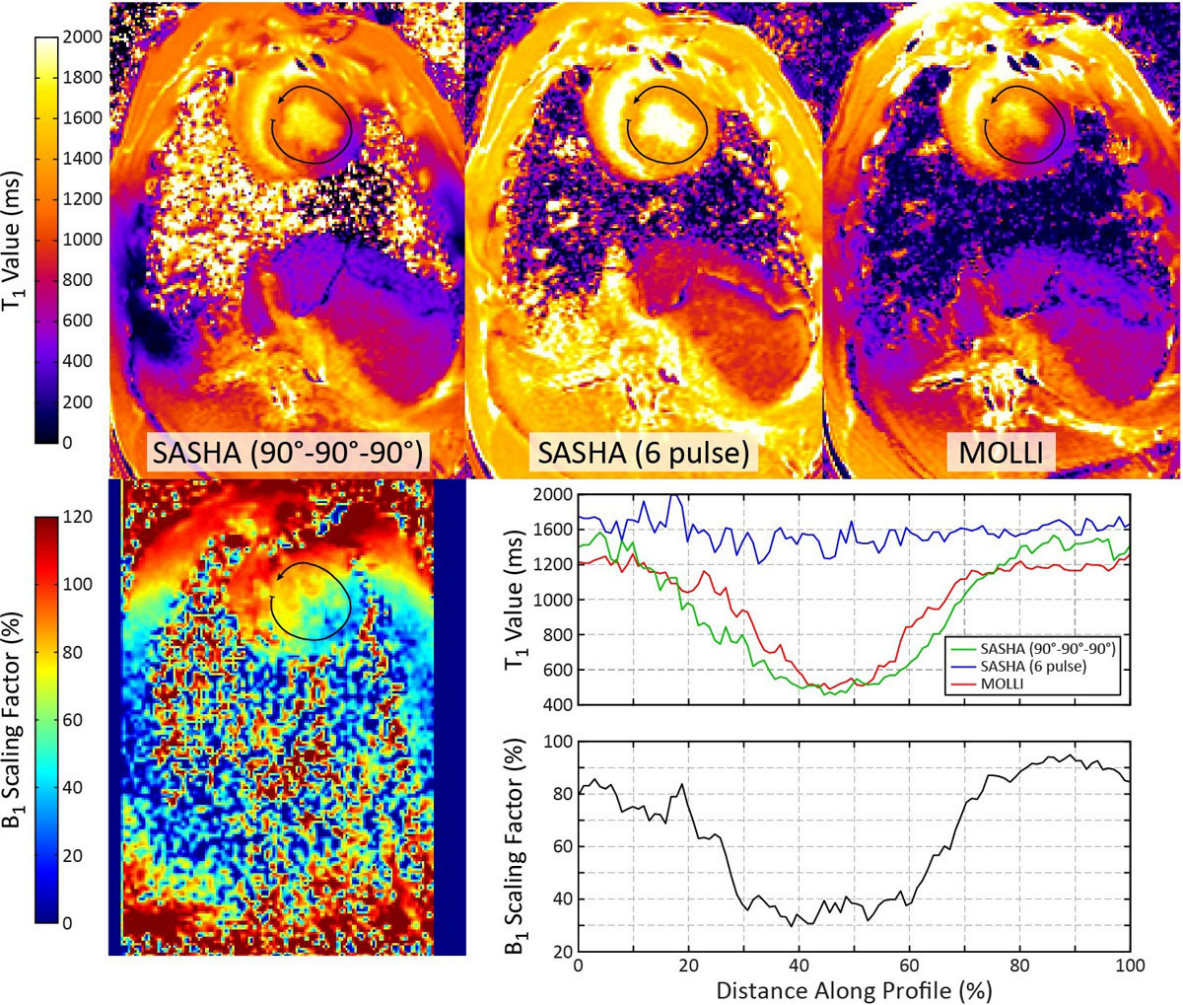
A B_1_ map (bottom left) and T_1_ maps (top row) using SASHA with a reference 90°-90°-90° saturation pulse, SASHA with a proposed 6 pulse train, and MOLLI in a swine. A profile of T_1_ values along the left ventricular wall shows decreased T_1_ values in the lateral wall coinciding with reduced B_1_ values.

In the swine study, the B_1_ varied from 30-95% across the left ventricle (LV) profile (Fig. 2). MOLLI and 90°-90°-90° SASHA T_1_ maps show a >50% artifactual decrease in T_1_ values with reduced B_1_ values in the lateral wall. SASHA T_1_ values using the 6-pulse train are more spatially homogeneous (1386±70 ms across the entire LV profile).

## Conclusions

A saturation pulse train optimized for B_0_, B_1_, and T_1_ ranges expected at 3T was shown to have residual longitudinal magnetization of <1%. In-vivo swine MOLLI and SASHA data with the commonly used 90°-90°-90° pulses had >50% T_1_ variation due to B_1_ inhomogeneity while 6-pulse train SASHA had a 5% coefficient of variation.

## Funding

Canadian Institutes of Health Research, Alberta Innovates - Health Solutions, NIH/NHLBI Intramural Research Program.
